# Evaluation of renal microvascular perfusion in dogs with cardiac disease using Superb Microvascular Imaging

**DOI:** 10.1093/jvimsj/aalag198

**Published:** 2026-09-07

**Authors:** Risa Yasuda, Kensuke Nakamura, Noboru Sasaki, Nozomu Yokoyama, Kazuyoshi Sasaoka, Mei Sugawara-Suda, Sei Kawamoto, Nozomi Shiohara, Yuki Kawakami, Kei Sato, Mitsuyoshi Takiguchi

**Affiliations:** Laboratory of Veterinary Internal Medicine, Department of Veterinary Clinical Sciences, Graduate School of Veterinary Medicine, Hokkaido University, Sapporo 060-0818, Japan; Laboratory of Veterinary Internal Medicine, Department of Veterinary Clinical Sciences, Graduate School of Veterinary Medicine, Hokkaido University, Sapporo 060-0818, Japan; Veterinary Teaching Hospital, Faculty of Veterinary Medicine, Hokkaido University, Sapporo 060-0818, Japan; Laboratory of Veterinary Internal Medicine, Department of Veterinary Clinical Sciences, Graduate School of Veterinary Medicine, Hokkaido University, Sapporo 060-0818, Japan; Veterinary Teaching Hospital, Faculty of Veterinary Medicine, Hokkaido University, Sapporo 060-0818, Japan; Veterinary Teaching Hospital, Faculty of Veterinary Medicine, Hokkaido University, Sapporo 060-0818, Japan; Veterinary Teaching Hospital, Faculty of Veterinary Medicine, Hokkaido University, Sapporo 060-0818, Japan; Laboratory of Veterinary Internal Medicine, Department of Veterinary Clinical Sciences, Graduate School of Veterinary Medicine, Hokkaido University, Sapporo 060-0818, Japan; Laboratory of Veterinary Internal Medicine, Department of Veterinary Clinical Sciences, Graduate School of Veterinary Medicine, Hokkaido University, Sapporo 060-0818, Japan; Laboratory of Veterinary Internal Medicine, Department of Veterinary Clinical Sciences, Graduate School of Veterinary Medicine, Hokkaido University, Sapporo 060-0818, Japan; Laboratory of Veterinary Internal Medicine, Department of Veterinary Clinical Sciences, Graduate School of Veterinary Medicine, Hokkaido University, Sapporo 060-0818, Japan

**Keywords:** cardiac disease, cardiorenal syndrome, congestion, dog, renal perfusion, ultrasonography

## Abstract

**Background:**

Renal dysfunction in cardiac disease is attributed to both reduced cardiac output and venous congestion; however, relative contributions of these mechanisms are difficult to evaluate clinically.

**Hypothesis/Objectives:**

Renal microvascular perfusion assessed by Superb Microvascular Imaging (SMI) would be associated with indices of cardiac output and venous congestion in dogs with cardiac disease.

**Animals:**

Forty-three client-owned dogs were enrolled: 15 healthy dogs (Control), 21 dogs with cardiac disease but without right-sided heart failure (non-RHF), and 7 dogs with signs of right-sided heart failure (RHF).

**Methods:**

Prospective cross-sectional observational study. SMI images were obtained from renal cortex, and mean SMI signal intensity (SMI-SI) was calculated as an index of renal perfusion. The left ventricular outflow tract velocity-time integral (LVOT-VTI) was used as an index of cardiac output, while caudal vena cava short-to-long axis ratio at minimal inspiratory diameter (CVC SD/LD) was used to indicate venous congestion.

**Results:**

Median SMI-SI was 41.3 (95% CI, 39.1-43.8) in Control, 21.6 (95% CI: 19.4-23.9) in non-RHF, and 18.6 (95% CI, 16.4-21.0) in RHF. Both cardiac disease groups had lower values than Control (*P* < .001). SMI-SI was negatively correlated with CVC SD/LD (ρ = −0.59, *P* < .001) and positively correlated with LVOT-VTI (ρ = 0.43, *P* = .007). In full multivariable analysis, only CVC SD/LD was independently associated with SMI-SI (*P* = .001).

**Conclusions and clinical importance:**

SMI detects reduced renal microvascular perfusion in dogs with cardiac disease, which is associated with venous congestion rather than cardiac output.

## Introduction

The heart and kidneys are closely interrelated, and dysfunction in one organ can lead to dysfunction in the other,^1^ referred to as cardiorenal syndrome. Traditionally, renal dysfunction associated with cardiac disease is attributed primarily to reduced renal perfusion caused by decreased cardiac output.^2^^,^^3^ However, recent evidence suggests that renal congestion caused by elevated caudal vena cava (CVC) pressure is also an important contributor to renal dysfunction.^4^ These alterations in renal hemodynamics in dogs with cardiac disease contribute to renal dysfunction and substantially affect clinical outcomes.^5^^,^^6^ Therefore, establishing reliable methods for early detection is critically important.

Clinically established methods for assessment of renal hemodynamic changes, such as hypoperfusion and congestion, remain limited. Although the glomerular filtration rate (GFR) can reflect renal blood flow, its measurement requires serial blood sampling over an extended period, limiting its clinical applicability.^7^ In practice, GFR can be indirectly estimated using blood biomarkers such as serum creatinine concentration or SDMA (symmetric dimethylarginine). However, because these biomarkers become abnormal only after a certain degree of renal function has been lost,^8^^,^^9^ their utility for detecting early renal hemodynamic changes might be limited. Furthermore, systemic hemodynamic variables that influence renal blood flow, such as cardiac output and CVC pressure, are considered standard reference measures when obtained by catheterization. Nevertheless, this approach is invasive and impractical for routine use.^10^ Importantly, these variables reflect systemic circulation and do not directly evaluate renal perfusion at the organ level. Therefore, non-invasive ultrasonographic techniques, such as Doppler ultrasonography^11^ and contrast-enhanced ultrasonography,^12^ have been investigated to evaluate renal perfusion at the organ level. However, Doppler ultrasonography might not detect subtle changes in renal perfusion during the subclinical phase of cardiac disease,^13^^,^^14^ and contrast-enhanced ultrasonography is not practical for routine clinical use.

Superb Microvascular Imaging (SMI) is a novel ultrasonographic technique that enables visualization of low-velocity blood flow within microvessels by suppressing low-frequency artifacts and providing a higher frame rate than conventional Doppler methods, without the need for contrast agents.^15^^,^^16^ We hypothesized that SMI would detect differences in renal microvascular perfusion among control dogs, dogs with cardiac disease, and dogs with right-sided heart failure. The objective of this study was to evaluate renal microvascular perfusion in dogs with cardiac disease using SMI and to investigate associations of SMI-derived renal perfusion indices with cardiac output and CVC indices.

## Materials and methods

This prospective cross-sectional observational study included client-owned dogs undergoing SMI at the Hokkaido University Veterinary Teaching Hospital between July 2023 and September 2024. All dogs were examined without sedation.

Dogs aged <7 years, with a serum creatinine concentration >1.4 mg/dL, or with systemic diseases were excluded.

**Figure 1:**
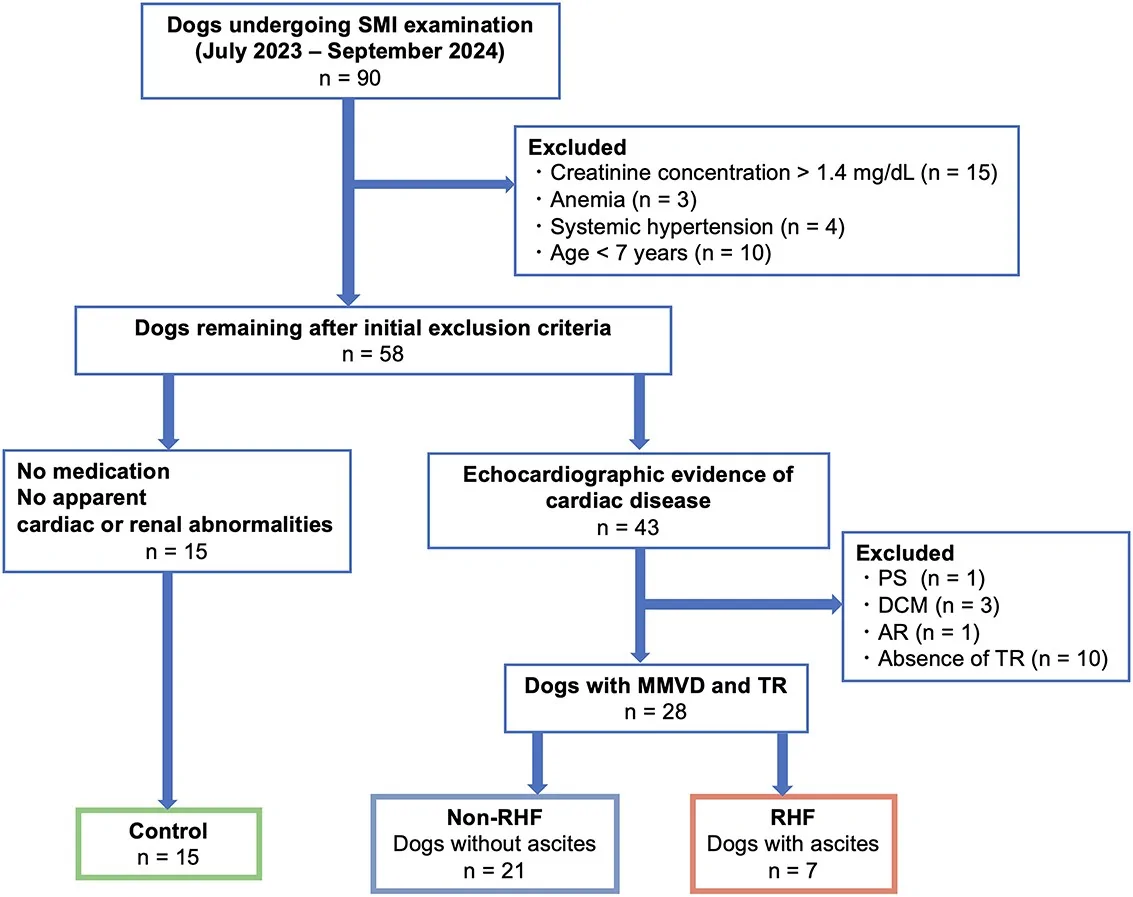
Flow diagram of study enrollment and group classification. Details of the inclusion and exclusion criteria are provided in the appendix. Abbreviations: AR = aortic regurgitation; DCM = dilated cardiomyopathy; MMVD = myxomatous mitral valve disease; PS = pulmonic stenosis; RHF = right-sided heart failure; SMI = Superb Microvascular Imaging; TR = tricuspid regurgitation.

Dogs receiving no medication and showing no cardiac or renal abnormalities on examination were classified as Control. Among dogs with echocardiographic evidence of cardiac disease, those with myxomatous mitral valve disease (MMVD) and tricuspid regurgitation were included. MMVD was staged according to the ACVIM consensus guidelines.^17^ Pulmonary hypertension (PH) was diagnosed when ACVIM criteria for high-probability PH were met.^18^ Dogs with ascites due to heart disease were classified as RHF, and those without ascites as non-RHF (Figure 1).

### Echocardiography

Echocardiographic examinations were performed using a Vivid E95 system (GE Healthcare Japan, Tokyo, Japan). Standard B-mode and Doppler imaging evaluated cardiac structure and regurgitation. The left ventricular outflow tract velocity-time integral (LVOT-VTI) was measured in the left apical 5-chamber view using pulsed-wave Doppler and used as a surrogate indicator of cardiac output.^19^

### Abdominal ultrasonography

Abdominal ultrasonography was performed using an Aplio i800 system (Canon Medical Systems, Tochigi, Japan). At minimal inspiratory diameter, the shortest and longest CVC diameters were measured to calculate the CVC short-to-long axis ratio (CVC SD/LD), used as an indicator of venous congestion.^20^

### Superb Microvascular Imaging measurement

**Figure 2:**
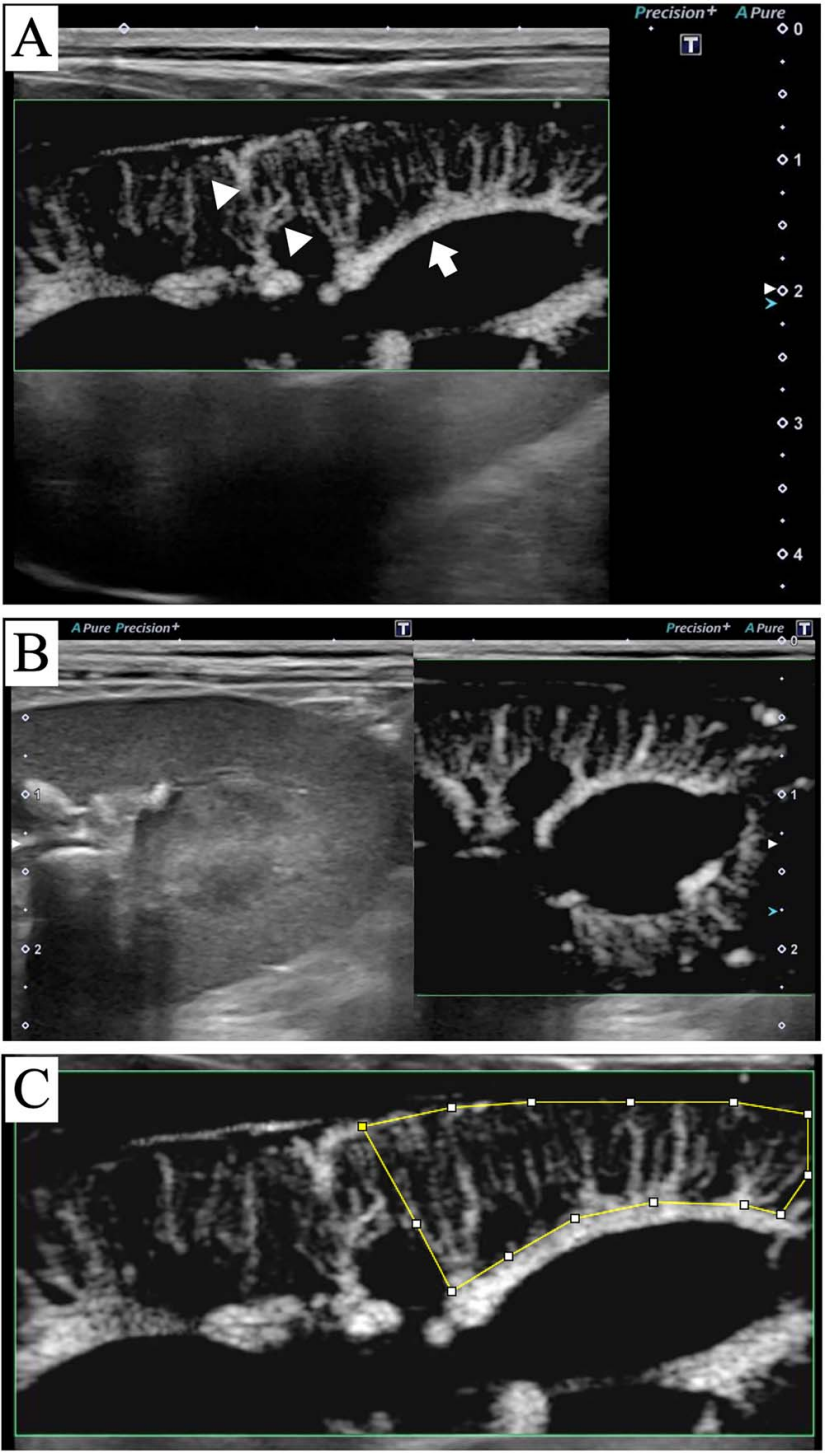
Representative monochrome Superb Microvascular Imaging (mSMI) images and region of interest (ROI) setting for SMI-SI (Superb Microvascular Imaging signal intensity) measurement. (A) mSMI image of the renal cortex. The arrow indicates an arcuate vessel, and the arrowheads indicate interlobular vessels. (B) Comparison of mSMI and B-mode images using the Twin View display. (C) Method for ROI placement in SMI-SI measurement. The polygonal outline indicates the manually drawn ROI.

SMI was performed with an Aplio i800 system using a linear transducer (i18LX5). Using monochrome SMI,^21^ cine loops of the left renal arcuate arteries were recorded, and frames with maximal blood flow signals were selected (Figure 2A). B-mode images were displayed simultaneously for anatomical reference (Figure 2B). A region of interest (ROI) was placed on the ventral renal cortex (Figure 2C). Images were converted to 8-bit grayscale (0-255), and the mean grayscale value was calculated.^22^ The average of 3 measurements was defined as SMI signal intensity (SMI-SI).

### Statistical analyses

Statistical analyses were performed using JMP® Pro 17.2. Continuous data are presented as median (IQR). For SMI-SI, 95% confidence intervals were bootstrapped. Groups were compared using Kruskal–Wallis with Dunn’s correction. Categorical variables were compared using Fisher’s exact test, and correlations were assessed using Spearman’s coefficient. A full multivariable regression model evaluated variables independently associated with SMI-SI. Significance was set at *P* < .05.

See Appendix for additional details.

## Results

A total of 43 dogs were enrolled in the study (15 Control, 21 non-RHF, and 7 RHF). The Control group included 4 Chihuahuas, 3 mixed-breed dogs, 2 Beagles, 2 Miniature Schnauzers, and 1 each of a Shetland Sheepdog, Yorkshire Terrier, Jack Russell Terrier, and Miniature Dachshund. The non-RHF group included 4 Chihuahuas, 4 mixed-breed dogs, 3 Miniature Schnauzers, 2 Toy Poodles, 2 Maltese, 2 Pomeranians, and 1 each of a Miniature Dachshund, Yorkshire Terrier, Boston Terrier, and Cavalier King Charles Spaniel. The RHF group included 2 mixed-breed dogs and one each of Chihuahua, Toy Poodle, Shih Tzu, Maltese, and Boston Terrier.

**Figure 3:**
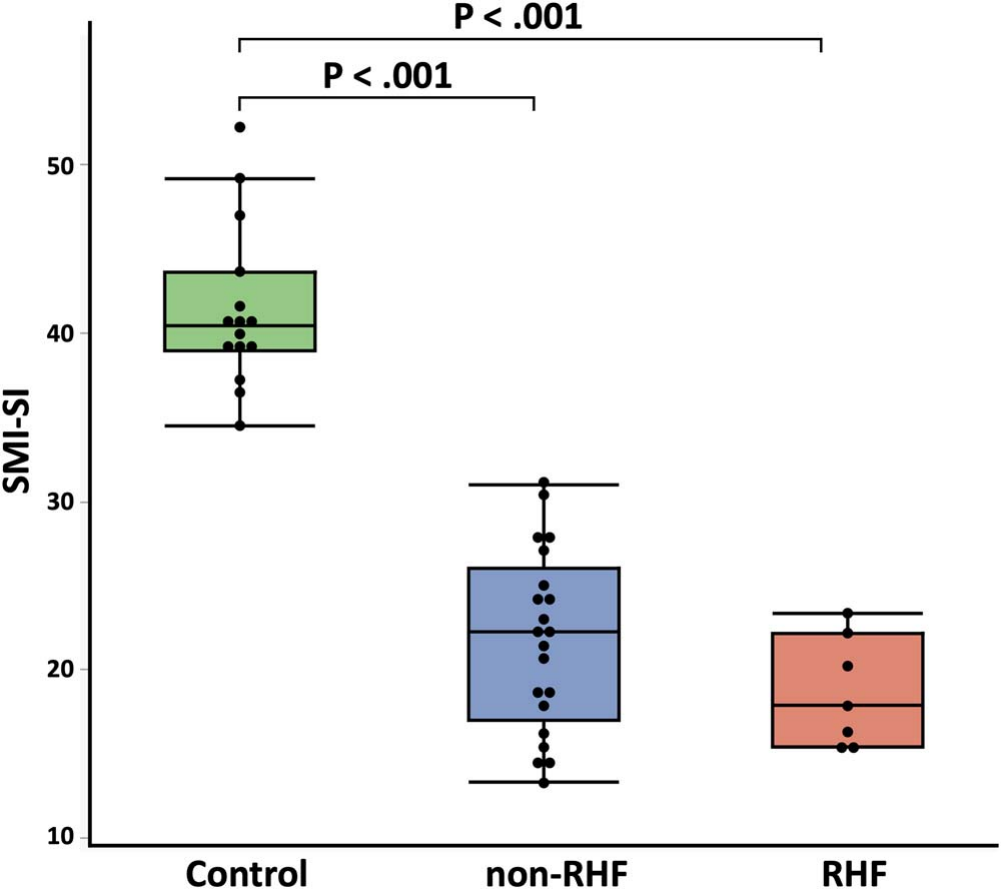
Box-and-whisker plots of SMI-SI (Superb Microvascular Imaging signal intensity) values among groups.

**Figure 4:**
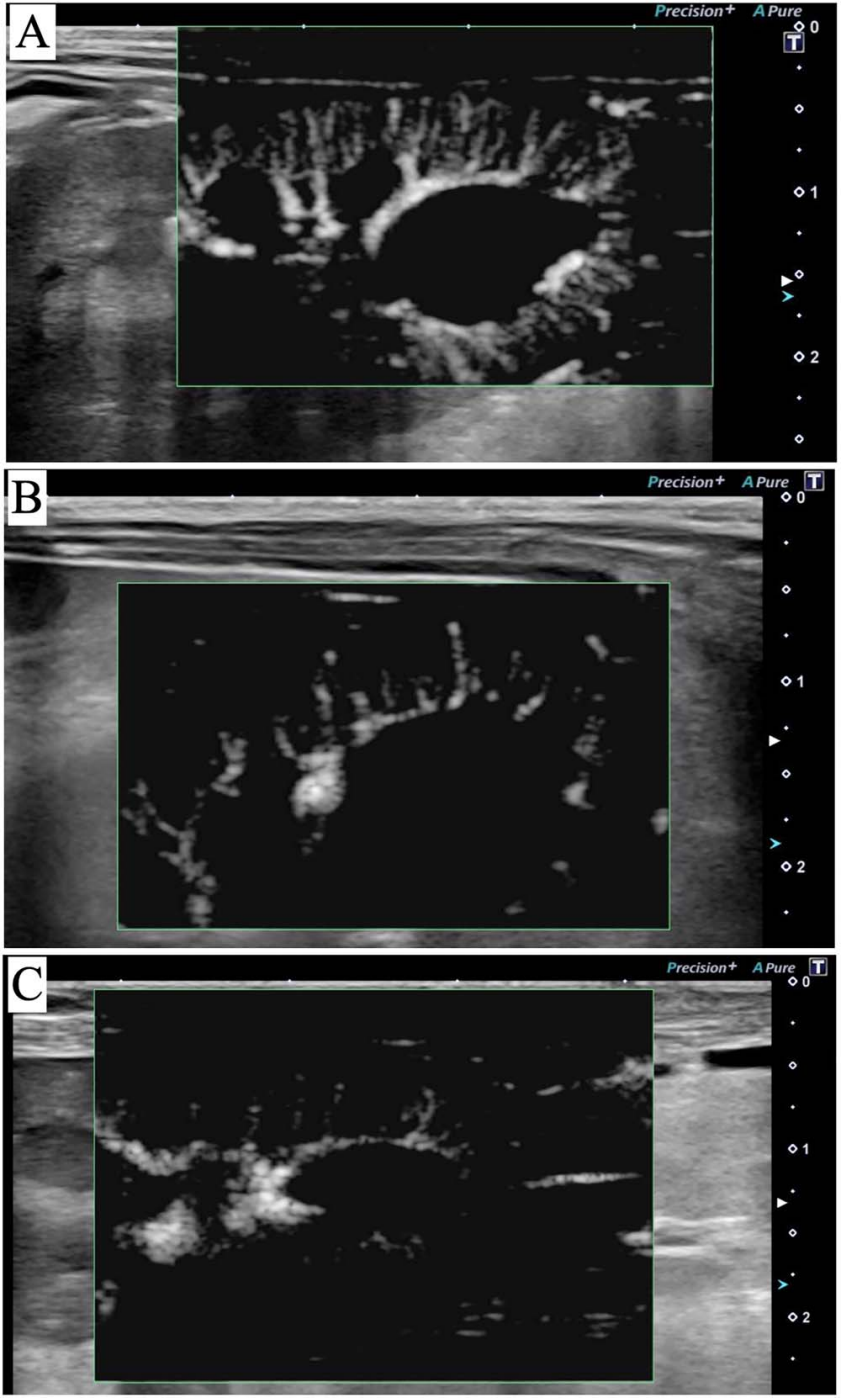
Representative SMI images showing progressively reduced cortical microvascular signals from control (A) to non-RHF (B) and RHF (C). Abbreviations: RHF = right-sided heart failure; SMI = Superb Microvascular Imaging.

The signalment and results of clinical examinations for all cases are summarized in Table 1. SMI-SI, CVC SD/LD, and LVOT-VTI differed significantly among the 3 groups (Kruskal–Wallis test, all *P* < .05); therefore, post hoc pairwise comparisons were performed for each variable. The median SMI-SI was 41.3 (95% CI, 39.1-43.8) in Control, 21.6 (95% CI, 19.4-23.9) in non-RHF, and 18.6 (95% CI, 16.4-21.0) in RHF. Compared with Control, SMI-SI values were significantly lower in both non-RHF and RHF groups (*P* < .001; Figures 3 and 4). CVC SD/LD ratios were 0.28 (0.25-0.30) in Control, 0.48 (0.34-0.64) in non-RHF, and 0.79 (0.48-0.87) in RHF. RHF showed significantly higher values than Control (*P* < .001), whereas non-RHF also differed significantly from Control (*P* = .004). LVOT-VTI values were 13.0 (11.1-13.9) cm in Control, 8.7 (8.2-12.3) cm in non-RHF, and 9.1 (6.5-11.7) cm in RHF. LVOT-VTI was significantly lower in the RHF group than in the Control group (*P* = .03), whereas the difference between the non-RHF and Control groups was not significant (*P* = .13). SMI-SI did not differ significantly among the cardiac disease classification groups (*P* = .53).

**Table 1:** Characteristics of the study population.

	Control	Non-RHF	RHF	*P*
***n***	15	21	7	
**Sex (male/female)**	9/6	6/15	4/3	.12
**Age (years)**	10 (8–12)	12 (9.5–13)	12 (7–13)	.47
**Body weight (kg)**	6.2 (4.3–10.0)	4.6 (3.4–6.7)	5.2 (3.5–9.6)	.06
**Creatinine concentration (mg/dL)**	0.6 (0.5–1)	0.9 (0.7–1.2)	0.9 (0.7–1.0)	.06
**Medication use (*n*)**				
** No medication use**	15	7	2	<.001
** Pimobendan**	−	13	3	.42
** ACE inhibitors**	−	5	2	1
** Loop diuretics**	−	7	2	1
** Spironolactone**	−	4	1	1
** Sildenafil**	−	1	2	.15
**Cardiac disease classification (*n*)**				.01
** PH**	−	7	7	
** MMVD stage B2 without PH**	−	5	0	
** MMVD stage C without PH**	−	9	0	
**Peak TRV (m/s)**	−	3.2 (2.5-3.5)	3.8 (3.0-4.0)	.04

Values are presented as median (IQR). *P*-values compare all 3 groups unless otherwise specified. *P*-values for individual medications compare the non-RHF and RHF groups only. The *P*-value for cardiac disease classification represents the overall comparison of disease-classification distribution between the non-RHF and RHF groups. Abbreviations: ACE inhibitors = angiotensin-converting enzyme inhibitors; MMVD = myxomatous mitral valve disease; PH = pulmonary hypertension; TRV = tricuspid regurgitation velocity.

**Figure 5:**
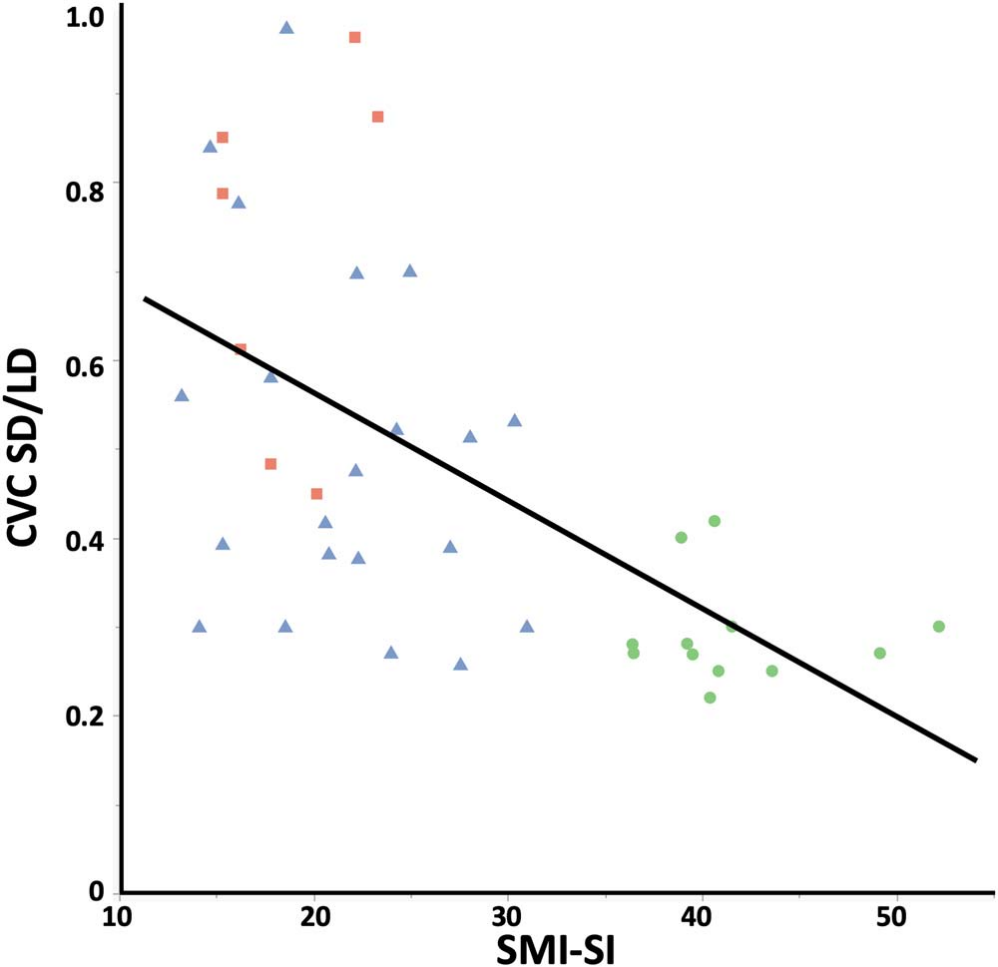
Scatter plot of SMI-SI versus CVC SD/LD. SMI-SI showed a strong negative correlation with CVC SD/LD (ρ = −0.59, *P* < .001). Green circles, blue triangles, and red squares represent the control, non-RHF, and RHF groups, respectively. Abbreviations: CVC SD/LD = caudal vena cava short-diameter-to-long-diameter ratio; RHF = right-sided heart failure; SMI-SI = Superb Microvascular Imaging signal intensity.

**Figure 6:**
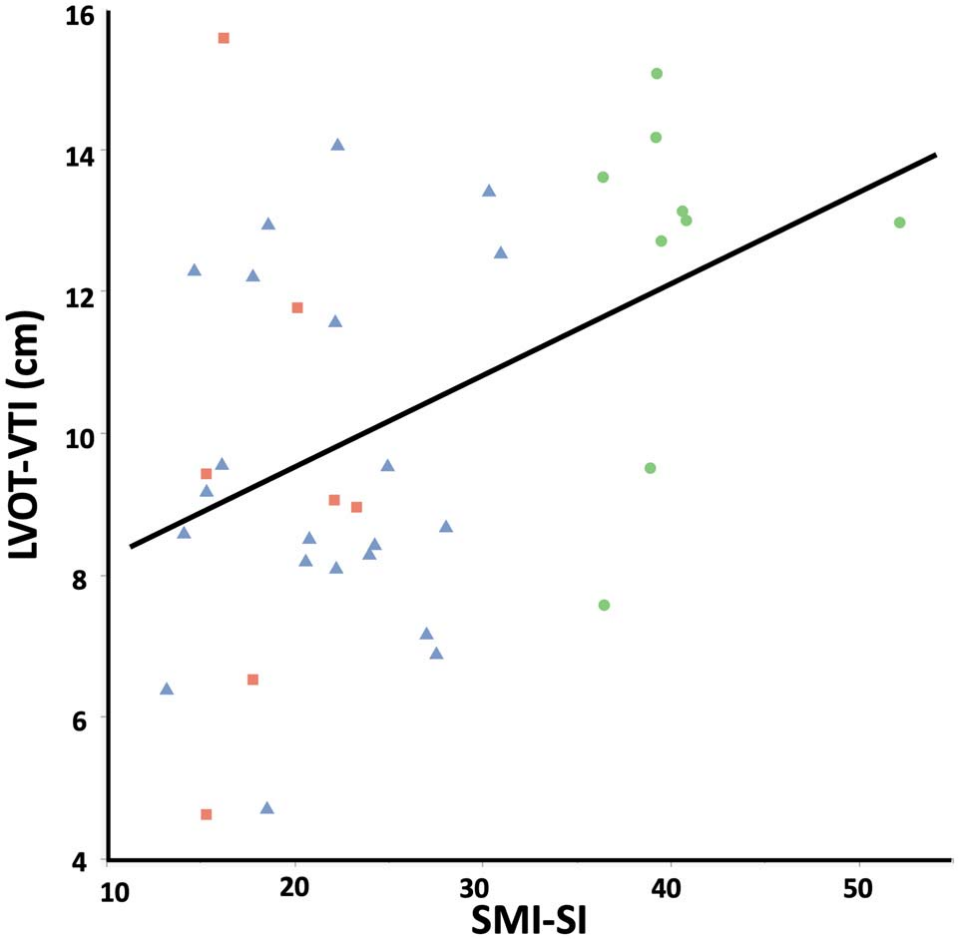
Scatter plot of SMI-SI versus LVOT-VTI. SMI-SI was positively correlated with LVOT-VTI (ρ = 0.43, *P* = .007). Green circles, blue triangles, and red squares represent the control, non-RHF, and RHF groups, respectively. Abbreviations: LVOT-VTI = left ventricular outflow tract velocity-time integral; RHF = right-sided heart failure; SMI-SI = Superb Microvascular Imaging signal intensity.

A negative correlation was observed between SMI-SI and CVC SD/LD (ρ = −0.59, *P* < .001), whereas a positive correlation was observed between SMI-SI and LVOT-VTI (ρ = 0.43, *P* = .007; Figures 5 and 6). In the full multivariable regression model, CVC SD/LD was the only variable independently associated with SMI-SI after adjustment for LVOT-VTI, body weight, age, and serum creatinine concentration (Table 2). Variance inflation factors did not indicate substantial multicollinearity. Residual diagnostic plots showed no major violations of model assumptions, and the residuals did not significantly deviate from normality based on the Shapiro–Wilk test (*P* = .87). Model fit is summarized in Table 2.

**Table 2:** Full multivariable regression analysis of variables associated with SMI-SI.

Variable	Regression coefficient (95% CI)	*P*	VIF
**CVC SD/LD**	−21.82 (−34.05 to −9.58)	.001	1.05
**LVOT-VTI**	0.97 (−0.06 to 2.01)	.06	1.24
**Body weight**	0.71 (−0.43 to 1.85)	.22	1.51
**Age**	−0.45 (−1.60 to 0.70)	.43	1.05
**Creatinine concentration**	−1.19 (−11.75 to 9.37)	.82	1.35

Model statistics: *n* = 36, adjusted *R*^2^ = 0.38, RMSE = 7.62, overall model *P* = .001.

Abbreviations: CVC SD/LD = caudal vena cava short-diameter-to-long-diameter ratio; LVOT-VTI = left ventricular outflow tract velocity-time integral; RMSE = root mean square error; SMI-SI = Superb Microvascular Imaging signal intensity; VIF = variance inflation factor.

## Discussion

In this study, SMI-SI detected significant reductions in renal microvascular perfusion in dogs with cardiac disease and these changes were independently associated with an index of venous congestion. SMI-SI was markedly lower in dogs with cardiac disease. Previously, reductions in renal perfusion associated with cardiac disorders have been demonstrated only through invasive assessments, such as GFR measurement or microsphere analysis, in experimentally induced models, including rapid ventricular pacing and abdominal aorta-CVC shunt models.^23^^,^^24^ This study demonstrates a similar reduction in renal blood flow in clinical cases of cardiac disease in dogs using a noninvasive imaging technique. In addition, the present study suggested that SMI-SI was strongly associated with CVC pressure estimated by CVC SD/LD. Elevation of right atrial pressure leads to increases in CVC and renal venous pressures, resulting in renal congestion. This congestion has been reported to cause medullary edema and compression of the peritubular capillaries, thereby reducing renal blood flow.^25^ SMI might therefore reflect such reductions in renal blood flow.

Among the methods used to assess renal congestion in patients with cardiac disease, pulsed-wave Doppler analysis of intrarenal venous flow (IRVF) is well known.^13^^,^^14^ However, more than half of patients with heart failure show no alterations in IRVF,^14^ and similar findings are reported in dogs.^13^ In contrast, a marked reduction in SMI-SI was observed even in dogs with cardiac disease that had not yet developed heart failure. Taken together, these findings suggest that while IRVF reflects changes in renal blood flow at advanced stages of cardiac disease, SMI-SI could detect alterations in renal perfusion at earlier stages. Unlike IRVF, which focuses on relatively large vessels such as interlobar veins entering and exiting the kidney, SMI visualizes blood flow in smaller vessels, including interlobular arteries that are distributed throughout the renal parenchyma. These anatomical differences in the observed vessels might therefore account for the distinct information provided by each method.

Similar to SMI, contrast-enhanced ultrasonography (CEUS) can also visualize blood flow within small vessels, such as interlobular arteries that are distributed throughout the renal parenchyma. In dogs with MMVD stage B2, CEUS shows significantly lower contrast enhancement compared with healthy dogs, indicating that CEUS can detect renal perfusion alterations in dogs with cardiac disease before the onset of heart failure.^12^ Furthermore, dogs with reduced right ventricular function exhibit even lower contrast enhancement; however, the relationship between this finding and renal congestion remains unclear. In contrast, although the association between SMI-SI and right ventricular function remains unclear, the present study suggested a relationship between SMI-SI and renal congestion. Because impaired right ventricular function might contribute to renal congestion,^26^ these 2 imaging modalities might reflect similar pathophysiological conditions. Given that SMI can detect renal perfusion abnormalities comparable to those identified by CEUS without the use of contrast agents, it could represent a valuable tool for clinical application.

In terms of practical use, SMI might have advantages over pulsed-wave Doppler and CEUS. SMI images can be acquired by visualizing the kidney in SMI mode for only a few seconds, and operators who are able to perform routine abdominal ultrasonography could acquire these images without extensive additional training. In contrast, pulsed-wave Doppler is angle-dependent and requires appropriate probe positioning and angle correction, whereas CEUS requires contrast administration and continuous imaging of the same region for a relatively long period. These features could support the clinical applicability of SMI as a simple and minimally invasive method for assessing renal microvascular perfusion.

In the present study, although both CVC SD/LD and LVOT-VTI remained within the normal range, a subset of dogs exhibited markedly lower SMI-SI values. This finding suggests the presence of factors other than systemic circulation associated with renal blood flow. For instance, local regulation of renal perfusion by neurohumoral mechanisms such as the renin–angiotensin–aldosterone system might contribute to reduced renal blood flow,^27^ and SMI-SI could be sensitive to renal microvascular changes related to such mechanisms.

This study has several limitations. In dogs with cardiac disease, a high serum creatinine concentration might result from cardiac disease-related hemodynamic alterations or from concurrent primary renal disease, such as chronic kidney disease, and distinguishing between these causes can be difficult. To minimize the influence of concurrent chronic kidney disease, dogs with high serum creatinine concentrations were excluded from the present study. However, this criterion might have excluded dogs with established cardiorenal syndrome. The GFR was not measured, and actual renal blood flow remains unknown. In addition, central venous pressure and cardiac output were estimated using echocardiographic indices, which are less accurate than invasive measurements. Furthermore, renal blood flow is known to be influenced by various factors. Although systematic data are lacking in dogs, studies in humans have identified age, sex, obesity, hydration status, and medication use as potential contributing factors.^28^ Although an inclusion criterion regarding age was established in the present study, complete standardization of these factors was not possible because of the clinical nature of the study. Therefore, it cannot be ruled out that noncardiac factors influenced renal blood flow and affected the results. Future studies should include a more comprehensive evaluation incorporating GFR and neurohumoral factors.

In conclusion, SMI-SI detected reductions in renal microvascular perfusion associated with cardiac disease. The findings suggested that renal congestion might be associated with SMI-SI.

## Off-label antimicrobial declaration

Authors declare no off-label use of antimicrobials.

## Institutional animal care and use committee or other approval declaration

Approved by the Clinical Research Ethics Committee of the Veterinary Teaching Hospital, Hokkaido University (Approval No. CR-23-016). Owner consent was obtained for all dogs prior to inclusion in the study.

## Appendix

### Materials and methods

This prospective cross-sectional observational study included client-owned dogs undergoing SMI at the Hokkaido University Veterinary Teaching Hospital between July 2023 and September 2024. All dogs underwent auscultation, systolic blood pressure measurement, thoracic radiography, abdominal ultrasonography, complete blood count, and serum biochemistry, including serum creatinine concentration. All examinations were performed without sedation. An a priori sample size calculation was not performed; instead, the sample size was determined by the number of dogs eligible for inclusion during the predefined study period.

Dogs were excluded if they had a serum creatinine concentration >1.4 mg/dL, were aged <7 years, or had systemic diseases. Systemic diseases included systemic hypertension, defined as systolic blood pressure >160 mmHg, and anemia, defined as hematocrit <35%.

Dogs were assigned to either the Control group or the cardiac disease group. The Control group consisted of dogs that were not receiving any medication at the time of examination and showed no apparent structural or functional abnormalities of the heart or kidneys, as assessed by auscultation, thoracic radiography, and abdominal ultrasonography. Specifically, Control dogs had no heart murmur or arrhythmia on auscultation, a vertebral heart size < 10.5 v on thoracic radiography, and normal renal structure on abdominal ultrasonography.

The cardiac disease group consisted of dogs with echocardiographic evidence of myxomatous mitral valve disease (MMVD) and tricuspid regurgitation. Dogs with congenital heart disease or cardiomyopathy were excluded to minimize potential confounding effects related to differing hemodynamic mechanisms. MMVD was staged according to the ACVIM consensus guidelines.^17^ Pulmonary hypertension (PH) was diagnosed when ACVIM criteria for high-probability PH were met.^18^

In dogs with cardiac disease, the presence or absence of ascites, a clinical sign of right-sided heart failure, was assessed. When ascites was present, abdominal fluid analysis was performed to confirm modified transudate, and biochemical testing and imaging studies were conducted to exclude causes other than cardiac disease. Dogs without ascites were classified as the non-RHF group, whereas dogs with ascites were classified as the RHF group. Case selection and group allocation are shown in Figure 1.

### Echocardiography

Echocardiographic examinations were performed using a Vivid E95 ultrasound system (GE Healthcare Japan, Tokyo, Japan) equipped with a sector probe (6S). To ensure consistency, all dogs were examined by, or under the direct supervision of, a Diplomate of the Asian College of Veterinary Internal Medicine (AiCVIM). All dogs were examined without sedation and positioned in right and left lateral recumbency, with simultaneous recording of a lead II electrocardiogram. The heart was assessed in B-mode, and color Doppler imaging was used to evaluate the presence of regurgitation. When regurgitation was detected, regurgitant jet velocity was obtained using continuous-wave Doppler. In the left apical 5-chamber view, the pulsed-wave Doppler sample gate was placed at the aortic annulus within the left ventricular outflow tract, and the outer edge of the spectral Doppler envelope was traced to measure the LVOT-VTI. LVOT-VTI was used as a surrogate indicator of cardiac output.^19^

### Abdominal ultrasonography

Abdominal ultrasonography was performed using an Aplio i800 ultrasound system (Canon Medical Systems, Tochigi, Japan) using a convex transducer (i8CX1). All abdominal ultrasonographic examinations were performed, stored, and reviewed by a single operator under the direct supervision of a Diplomate of the AiCVIM to ensure consistency. All dogs were examined without sedation and positioned in dorsal recumbency to evaluate the presence or absence of ascites and structural abnormalities of the kidneys. In addition, the probe was placed at the right intercostal space to obtain a transverse view of the hepatic hilum, and a transverse image of the CVC was acquired. At the time of minimal CVC diameter during inspiration, the shortest diameter (SD) and longest diameter (LD) of the CVC were measured, and the CVC short-to-long axis ratio (CVC SD/LD) was calculated. The CVC SD/LD was used as an indicator of venous congestion, which may contribute to renal congestion.^20^

### Superb Microvascular Imaging (SMI) measurement

SMI was performed with an Aplio i800 ultrasound system using a linear transducer (i18LX5). All dogs were examined without sedation and positioned in dorsal recumbency. In SMI mode, the arcuate arteries on the ventral aspect of the left kidney were visualized and recorded as cine loops. SMI provides 2 display modes: color-coded SMI (cSMI) and monochrome SMI (mSMI). The cSMI mode allows simultaneous display of color-coded Doppler signals over grayscale B-mode images. In contrast, the mSMI mode suppresses background information and displays microvascular blood flow in grayscale, providing higher sensitivity for detecting low-velocity flow than cSMI.^21^ Based on these characteristics, mSMI was used in the present study.

mSMI images were acquired as shown in Figure 2A. When appropriate, B-mode images were displayed simultaneously using the Twin View display for anatomical reference (Figure 2B). All measurements were performed in the mSMI mode using consistent settings: a frame rate ≥ 40 frames per second, a velocity range ± 2.3 cm/s, and a transmit/receive frequency setting of 6.0/11 MHz. The imaging depth was adjusted to confirm that the arcuate vessels were located within 2 cm from the transducer. For each dog, 3 cine loops were recorded, and from each cine loop, one frame demonstrating the maximum blood flow signal was visually selected for analysis. At the level of the arcuate vessels, an ROI was placed on the ventral renal cortex, covering at least one-fourth of the ventral interlobular vessel region (Figure 2C). The acquired images were converted to 8-bit grayscale (0-255), and the mean grayscale value of all pixels within the ROI was calculated.^22^ Measurements were performed in 3 representative frames per dog with optimal image quality, and the mean value was defined as SMI-SI. All image analyses were performed using commercially available software (ImageJ 1.53 t; Wayne Rasband and contributors, National Institutes of Health, Bethesda, MD, USA). All SMI cine loops were acquired and stored by a single operator. After image acquisition, all SMI-SI analyses were performed offline by the same operator using the stored cine loops. The operator was aware of the clinical status of the dogs at the time of image acquisition and analysis; therefore, blinding was not performed.

### Statistical analyses

Statistical analyses were performed using commercially available software (JMP® Pro 17.2, SAS Institute Inc., Cary, NC, USA). Continuous variables were summarized using medians and interquartile ranges. For SMI-SI, 95% CIs were estimated using bootstrap resampling, and the results are presented as medians with corresponding confidence intervals.

Differences in ultrasound parameters among the Control, non-RHF, and RHF groups were evaluated using the Kruskal–Wallis test. When significant, post hoc pairwise comparisons were performed using Dunn’s test with Bonferroni correction. In dogs with cardiac disease, differences in SMI-SI among the cardiac disease classification groups were evaluated using the Kruskal–Wallis test, and differences in SMI-SI between dogs with and without PH were evaluated using the Wilcoxon rank-sum test. Comparisons of categorical variables (eg, sex, medication, and disease distribution) among groups were analyzed using Fisher’s exact test. Correlations were assessed using Spearman’s correlation coefficient (ρ). The strength of the correlation was interpreted based on the absolute value of ρ.

Full multivariable regression analysis was conducted as a descriptive and exploratory analysis to evaluate variables associated with SMI-SI after adjustment for other candidate variables. Candidate explanatory variables were selected a priori based on their physiological or clinical relevance to renal microvascular perfusion. CVC SD/LD and LVOT-VTI were included as hemodynamic variables reflecting venous congestion and cardiac output, respectively. Serum creatinine concentration was included as a clinical indicator of renal function. Body weight and age were included as potential confounding variables that may influence renal vascular measurements independently of cardiovascular status. Given the limited sample size, the number of explanatory variables included in the multivariable model was restricted to reduce the risk of overfitting. Multicollinearity was assessed using variance inflation factors. Model assumptions were evaluated using residual diagnostic plots to assess linearity and homoscedasticity. The normality of residuals was assessed using residual diagnostic plots and the Shapiro–Wilk test. Model fit was assessed using adjusted *R*^2^ and root mean square error. The overall significance of the model was evaluated using the model F-test.

Statistical significance was set at *P* < .05.

## Abbreviations

ACE: angiotensin-converting enzyme
AR: aortic regurgitation
CEUS: contrast-enhanced ultrasonography
CI: confidence interval
cSMI: color-coded Superb Microvascular Imaging
CVC: caudal vena cava
CVC SD/LD: caudal vena cava short-to-long axis ratio
DCM: dilated cardiomyopathy
GFR: glomerular filtration rate
IRVF: intrarenal venous flow
LVOT-VTI: left ventricular outflow tract velocity-time integral
mSMI: monochrome Superb Microvascular Imaging
MMVD: myxomatous mitral valve disease
PH: pulmonary hypertension
PS: pulmonic stenosis
RHF: right-sided heart failure
ROI: region of interest
SDMA: symmetric dimethylarginine
SMI: Superb Microvascular Imaging
SMI-SI: Superb Microvascular Imaging signal intensity
TRV: tricuspid regurgitation velocity

## References

[1] Rangaswami J, Bhalla V, Blair JEA, et al. Cardiorenal syndrome: classification, pathophysiology, diagnosis, and treatment strategies: a scientific statement from the American Heart Association. Circulation. 2019;139:e840–e878. 10.1161/CIR.0000000000000664 30852913

[2] Ljungman S, Laragh JH, Cody RJ. Role of the kidney in congestive heart failure: relationship of cardiac index to kidney function. Drugs. 1990;39:10–21. 10.2165/00003495-199000394-00004 2354670

[3] Merrill AJ . Edema and decreased renal blood flow in patients with chronic congestive heart failure: evidence of “forward failure” as the primary cause of edema. J Clin Invest. 1946;25:389–400. 10.1172/JCI101720 20986045

[4] Mullens W, Abrahams Z, Francis GS, et al. Importance of venous congestion for worsening of renal function in advanced decompensated heart failure. J Am Coll Cardiol. 2009;53:589–596. 10.1016/j.jacc.2008.05.068 19215833 PMC2856960

[5] Martinelli E, Locatelli C, Bassis S, et al. Preliminary investigation of cardiovascular–renal disorders in dogs with chronic mitral valve disease. J Vet Intern Med. 2016;30:1612–1618. 10.1111/jvim.14524 27717188 PMC5032878

[6] Damman K, Voors AA, Hillege HL, et al. Congestion in chronic systolic heart failure is related to renal dysfunction and increased mortality. Eur J Heart Fail. 2010;12:974–982. 10.1093/eurjhf/hfq118 20685688

[7] Cobrin AR, Blois SL, Kruth SA, Abrams-Ogg ACG, Dewey C. Biomarkers in the assessment of acute and chronic kidney diseases in the dog and cat. J Small Anim Pract. 2013;54:647–655. 10.1111/jsap.12150 24152019

[8] McKenna M, Pelligand L, Elliott J, Cotter D, Jepson R. Relationship between serum iohexol clearance, serum SDMA concentration, and serum creatinine concentration in non-azotemic dogs. J Vet Intern Med. 2020;34:186–194. 10.1111/jvim.15659 31725186 PMC6979102

[9] Pelander L, Häggström J, Larsson A, et al. Comparison of the diagnostic value of symmetric dimethylarginine, cystatin C, and creatinine for detection of decreased glomerular filtration rate in dogs. J Vet Intern Med. 2019;33:630–639. 10.1111/jvim.15445 30791142 PMC6430914

[10] Teja B, Bosch NA, Diep C, et al. Complication rates of central venous catheters: a systematic review and meta-analysis. JAMA Intern Med. 2024;184:474–482. 10.1001/jamainternmed.2023.8232 38436976 PMC12285596

[11] Qian X, Zhen J, Meng Q, Li L, Yan J. Intrarenal Doppler approaches in hemodynamics: a major application in critical care. Front Physiol. 2022;13:951307. 10.3389/fphys.2022.951307 36311236 PMC9597190

[12] Kamata S, Morita T, Yamasaki M. Detection of early renal perfusion changes by contrast-enhanced ultrasonography in dogs with preclinical myxomatous mitral valve disease. Vet Radiol Ultrasound. 2025;66:e13459. 10.1111/vru.13459 39531152 PMC11617612

[13] Terukina H, Morita T, Kobayashi S, Uchida N, Yamasaki M. Renal venous doppler waveforms are altered by right-sided cardiac influences in dogs with heart disease. In: Proceedings of the 114th Annual Meeting of the Japanese Society of Veterinary Cardiology; 2021; Tokyo, Japan. Abstract C-17.

[14] Iida N, Seo Y, Sai S, et al. Clinical implications of intrarenal hemodynamic evaluation by Doppler ultrasonography in heart failure. JACC Heart Fail. 2016;4:674–682. 10.1016/j.jchf.2016.03.016 27179835

[15] Gao J, Thai A, Erpelding T. Comparison of superb microvascular imaging to conventional color Doppler ultrasonography in depicting renal cortical microvasculature. Clin Imaging. 2019;58:90–95. 10.1016/j.clinimag.2019.06.011 31284178

[16] Tang K, Liu M, Zhu Y, Zhang M, Niu C. The clinical application of ultrasonography with superb microvascular imaging: a review. J Clin Ultrasound. 2022;50:721–732. 10.1002/jcu.23210 35358353

[17] Keene BW, Atkins CE, Bonagura JD, et al. ACVIM consensus guidelines for the diagnosis and treatment of myxomatous mitral valve disease in dogs. J Vet Intern Med. 2019;33:1127–1140. 10.1111/jvim.15488 30974015 PMC6524084

[18] Reinero C, Visser LC, Kellihan HB, et al. ACVIM consensus statement guidelines for the diagnosis, classification, treatment, and monitoring of pulmonary hypertension in dogs. J Vet Intern Med. 2020;34:549–573. 10.1111/jvim.15725 32065428 PMC7097566

[19] Lopes PCF, Sousa MG, Camacho AA, et al. Comparison between two methods for cardiac output measurement in propofol-anesthetized dogs: thermodilution and Doppler. Vet Anaesth Analg. 2010;37:401–408. 10.1111/j.1467-2995.2010.00552.x 20712606

[20] Fujioka T, Nakamura K, Minamoto T, Tsuzuki N, Yamaguchi J, Hidaka Y. Ultrasonographic evaluation of the caudal vena cava in dogs with right-sided heart disease. J Vet Cardiol. 2021;34:80–92. 10.1016/j.jvc.2021.01.005 33626419

[21] Fu Z, Zhang J, Lu Y, et al. Clinical applications of superb microvascular imaging in the superficial tissues and organs: a systematic review. Acad Radiol. 2021;28:694–703. 10.1016/j.acra.2020.03.032 32418782

[22] Nakamura K, Sasaki N, Yoshikawa M, et al. Quantitative contrast-enhanced ultrasonography of canine spleen. Vet Radiol Ultrasound. 2009;50:104–108. 10.1111/j.1740-8261.2008.01499.x 19241764

[23] Yoshimura A, Ohmori T, Yamada S, et al. Comparison of pancreatic and renal blood flow in a canine tachycardia-induced cardiomyopathy model. J Vet Med Sci. 2020;82:836–845. 10.1292/jvms.19-0694 32336699 PMC7324827

[24] Westenfelder C, Arruda J, Lockwood R, Boonjarern S, Nascimento L, Kurtzman N. Distribution of renal blood flow in dogs with congestive heart failure. Am J Physiol. 1976;230:537–542. 10.1152/ajplegacy.1976.230.2.537 1259034

[25] Doty JM, Saggi BH, Sugerman HJ, et al. Effect of increased renal venous pressure on renal function. J Trauma. 1999;47:1000–1003. 10.1097/00005373-199912000-00002 10608524

[26] Tabucanon T, Tang WHW. Right heart failure and cardiorenal syndrome. Cardiol Clin. 2020;38:185–202. 10.1016/j.ccl.2020.01.004 32284096 PMC7160202

[27] Riegger AJG, Liebau G. The renin-angiotensin-aldosterone system, antidiuretic hormone and sympathetic nerve activity in an experimental model of congestive heart failure in the dog. Clin Sci (Lond). 1982;62:465–469. 10.1042/cs0620465 7075144

[28] Alhummiany B, Sharma K, Buckley DL, Soe KK, Sourbron SP. Physiological confounders of renal blood flow measurement. MAGMA. 2024;37:565–582. 10.1007/s10334-023-01126-7 37971557 PMC11417086

